# Eradication of *Candida albicans* Biofilm Viability: In Vitro Combination Therapy of Cationic Carbosilane Dendrons Derived from 4-Phenylbutyric Acid with AgNO_3_ and EDTA

**DOI:** 10.3390/jof7070574

**Published:** 2021-07-18

**Authors:** Natalia Gómez-Casanova, Tania Lozano-Cruz, Juan Soliveri, Rafael Gomez, Paula Ortega, José Luis Copa-Patiño, Irene Heredero-Bermejo

**Affiliations:** 1 Department of Biomedicine and Biotechnology, Faculty of Pharmacy, University of Alcalá, 28871 Alcalá de Henares, Spain; natalia.gomezc@uah.es (N.G.-C.); juan.soliveri@uah.es (J.S.); josel.copa@uah.es (J.L.C.-P.); 2 Department of Organic and Inorganic Chemistry, Research Institute in Chemistry, University of Alcalá, “Andrés M. del Río” (IQAR), 28871 Alcalá de Henares, Spain; tania.lozano@uah.es (T.L.-C.); rafael.gomez@uah.es (R.G.); paula.ortega@uah.es (P.O.); 3 Institute “Ramón y Cajal” for Health Research (IRYCIS), University of Alcalá, 28034 Madrid, Spain; 4 Networking Research Center on Bioengineering, Biomaterials and Nanomedicine (CIBER-BBN), 28029 Madrid, Spain

**Keywords:** *Candida albicans*, carbosilane dendron, silver nitrate, EDTA, biofilm, synergy

## Abstract

*Candida albicans* is a human pathogen of significant clinical relevance. This pathogen is resistant to different drugs, and most clinical antifungals are not effective against the prevention and treatment of *C. albicans* infections. As with other microorganisms, it can produce biofilms that serve as a barrier against antifungal agents and other substances, contributing to infection in humans and environmental tolerance of this microorganism. Thus, resistances and biofilm formation make treatment difficult. In addition, the complete eradication of biofilms in implants, catheters and other medical devices, is challenging and necessary to prevent relapses of candidemia. Therefore, it is a priority to find new molecules or combinations of compounds with anti-*Candida* biofilm activity. Due to the difficulty of treating and removing biofilms, the aim of this study was to evaluate the in vitro ability of different generation of cationic carbosilane dendrons derived from 4-phenylbutyric acid, ArCO_2_G_n_(SNMe_3_I)_m_, to eradicate *C. albicans* biofilms. Here, we assessed the antifungal activity of the second generation dendron ArCO2G_2_(SNMe_3_I)_4_ against *C. albicans* cells and established biofilms since it managed to seriously damage the membrane. In addition, the combinations of the second generation dendron with AgNO_3_ or EDTA eradicated the viability of biofilm cells. Alterations were observed by scanning electron microscopy and cytotoxicity was assessed on HeLa cells. Our data suggest that the dendritic compound ArCO_2_G_2_(SNMe_3_I)_4_ could represent an alternative to control the infections caused by this pathogen.

## 1. Introduction

*Candida* species are frequently found in the normal microbiota of humans. For this reason, it is common to find them in implanted biomaterials and medical instrumentation [1,2,3], being one of the main causes of catheter-related infections [4] and posing an important health risk for hospitalized patients when biofilm structures are formed [4,5]. The main problems associated to biofilm formation are their greater resistance to antifungal agents, the ability to withstand harsh conditions and the ability of biofilm cells to evade the host’s immune response [6]. Consequently, it represents a great challenge for patients with immunodeficiency or medical implanted devices, among others [7]. In addition, another problem lies in the fact that *Candida* is a eukaryotic microorganism, so the design of new molecules with antifungal activity is limited by the scarcity of targets and the tendency to present high cytotoxic levels [8]. Therefore, there is an urgent need to find alternative therapies to prevent and control *C. albicans* biofilm-related infections. In this sense, dendritic compounds have emerged as an interesting alternative in the field of biomedicine and could be a new therapeutic approach to combat the infections caused by this pathogen [9]. Dendritic systems have well defined and monodisperse structures and are being widely studied in various biomedical fields, such as drug delivery systems, antiviral or magnetic resonance imaging contrast agents, among others [10,11]. The properties of these dendrimeric systems such as monodispersity, multivalence, among others, give them the ability to act as drugs. Multivalence allows them to act as therapeutic agents, since the functional groups on the surface are those that interact with cell membranes. Another aspect is their solubility, which makes them of great interest at the clinical level. In their use as antibacterial agents, cationic dendritic molecules have been studied against different planktonic cells [12,13,14,15], and biofilms [9,16]. In addition, these molecules can overcome the problem of resistances to drugs because it has been reported that they do not induce antibiotic resistance in bacteria [17]. Furthermore, the synergistic combination of these structures with commercial drugs can improve the drug’s solubility and the antibacterial activity [14,18,19]. In particular, dendritic systems with a conical topology, called dendrons, make it possible to obtain systems with two therapeutic functions, one at the focal point and the other at the surface, in a precise and controlled manner to obtain a dual therapeutic action, for example two therapeutic fragments with different modes of action.

On the other hand, the treatment with different drugs in the so-called combination therapy is interesting from the point of view of reducing the doses to be administered, reducing the side effects of the drugs. Related to the use different compounds in combination therapy, silver is a compound with a low toxicity that is widely used in medicine [20,21]. Different salts such as silver nitrate (AgNO_3_), silver diamine fluoride or silver nanoparticles, among others; have a broad antibacterial and antifungal spectrum. In addition, their mechanics of action is consequence of their ability to bind and alter cell membranes, cellular respiration and DNA integrity [22]. Silver has been used in the treatment of wounds [23,24,25], in water purification [26], caries [21] and biofilms [27], along with other uses. Another compound, that has shown promising results in prevent [28,29,30] or treat established biofilms [30,31,32] is ethylenediaminetetraacetic acid (EDTA) either on its own or in combination with commercial antifungal drugs, significantly improving its antibiofilm activity.

In the present study, we investigated the antifungal activity of different generations cationic carbosilane dendrons derived from 4-phenylbutyric acid. In addition, the most active dendron was tested in combination with AgNO_3_ and EDTA to study their ability to prevent biofilm development or completely eradicate stablished *Candida albicans* biofilms. The morphological alterations caused by treatment were visualized by scanning electron microscopy (SEM), both in biofilm formation and established biofilms. Finally, the cytotoxicity of cationic carbosilane dendrons derived from 4-phenylbutyric acid were evaluated in a HeLa cell line.

## 2. Materials and Methods

### 2.1. Candida Albicans Strain and Culture Conditions

*Candida albicans* strain 1002 from Colección Española de Cultivos Tipo (CECT) were used in this study. *Candida* isolated was stored at −80 °C with 20% glycerol (Sigma-Aldrich, Saint Louis, MO, USA) until use. The strain was grown on Sabouraud chloramphenicol agar (Scharlab, Barcelona, Spain) overnight. To stimulate biofilm formation, various colonies were transferred into Yeast Extract (1%)–Peptone (2%)–Dextrose (2%) (YPD, Scharlab, Barcelona, Spain) and incubated at 37 °C with agitation (150 rpm) for 24 h.

### 2.2. Dendritic Compounds

**Figure 1:**
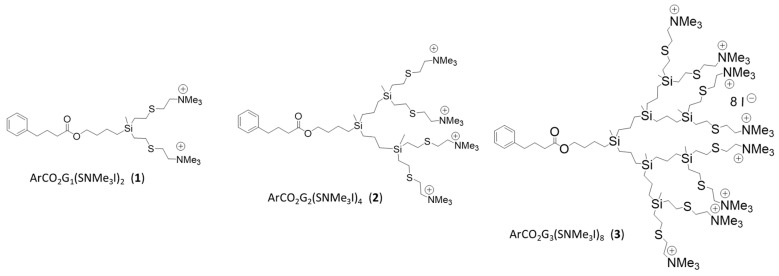
Structures of cationic carbosilane dendrons derived from 4-phenylbutyric acid ArCO_2_G_n_(SNMe_3_I)_m_ (n = 1; m = 2 (**1**), n = 2; m = 4 (**2**) and n = 3; m = 8 (**3**)) n: generation, m: number of functional groups.

Cationic carbosilane dendrons derived from 4-phenylbutyric acid (Sigma-Aldrich, Saint Louis, MO, USA) (Figure 1) soluble in water, were synthesized according to protocol described in literature [33]. Three generation of dendrons ArCO_2_G_n_(SNMe_3_I)_m_ (n = 1; m = 2 (**1**), n = 2; m = 4 (**2**) and n = 3; m = 8 (**3**)) were tested to study their in vitro ability to prevent biofilm formation and eliminate *C. albicans* established biofilms. The antifungal susceptibility testing was performed using the NCCLS M27A broth microdilution reference method [34,35]. Dendrons **1**–**3** were tested in 96-well microtiter plates using a series of two-fold dilutions with concentrations ranging from 2 to 512 mg/L against biofilm formation and from 2 to 1024 mg/L against established biofilms. Plates were incubated at 37 °C. Assays were run in technical triplicate and repeated at least twice in independent experiments. The data were expressed as arithmetic averages.

### 2.3. Antifungal and Antibiofilm Formation Susceptibility Test

The anti-biofilm activity of dendrons for inhibition of biofilm formation was performed as previously described [9]. An inoculum of *C. albicans* was adjusted to a density equivalent to 0.5 McFarland standard (1 × 10^6^ cells/mL, Grant Instruments, Royston, UK) in RPMI 1640 medium (Sigma-Aldrich) with morpholinepropanesulfonic acid (MOPS, Sigma-Aldrich) and 2% glucose (Scharlab, Barcelona, Spain) (RPMI + MOPS + GLU). Then, 50 μL of the suspension was inoculated in 96-well microtiter plates containing two-fold serial dilutions of the dendrons ranging from 2 to 512 mg/L. Controls were included in all experiments: un-inoculated medium and dendron free medium. Plates were sealed with Parafilm^®^ (Bemis, Neenah, WI, USA) and incubated for 48 h at 37 °C. Then, resazurin colorimetric assay (Section 2.6) and drop plate method (Section 2.7) were used to determine the minimum biofilm inhibitory concentration (MBIC) and the minimum fungicidal concentration (MFC), respectively. The MBIC was defined as the lowest concentration at which no growth (resazurin reduction) was observed [9]. The MFC was defined as the lowest concentration for which no growth was observed when plating 5 μL suspension of each well, and, in consequence, a biofilm was not formed. 

### 2.4. In Vitro Antibiofilm Susceptibility Test against Stablished Biofilms

In vitro biofilm formation was carried out as described above, and plates were incubated for 48 h at 37 °C until mature biofilms were formed. At the end of incubation, the medium was carefully aspirated, and biofilms were washed with sterile phosphate buffered saline (PBS, Sigma-Aldrich, Saint Louis, MO, USA) to remove non-adherent cells. Then, serial concentrations of dendritic compounds prepared in RPMI + MOPS + GLU were added to a final volume of 100 μL to study the antibiofilm activity (established biofilm). Controls were included in all experiments: un-inoculated medium and dendron free medium. Plates were sealed with Parafilm^®^ and incubated for 48 h at 37 °C. Then, resazurin colorimetric assay and drop plate method were used to determine the minimum biofilm damaging concentrations (MBDC) and the minimum biofilm eradicating concentration (MBEC), respectively. These assays determined the inhibition of established biofilms. The MBDC was defined as the lowest concentration that caused damage and affected or inhibited *Candida* metabolic activity. For these experiments, MBDC value has a similar meaning as the classic MIC, with the difference that these cells have developed a biofilm and we assume that most of them are damaged (detection by resazurin as non-viable, not absorbance signal detected). The MBEC was defined as the lowest concentration resulting in a 100% cell death of the biofilm (no growth observed on agar plates), therefore, biofilm cells were not viable [9,36].

### 2.5. Combination Therapy of Dendron ArCO_2_G_2_(SNMe_3_I)_4_ (**2**) with AgNO_3_ and EDTA against C. albicans

The potential synergistic activity was studied for the biofilm formation and established biofilm conditions using the checkerboard titration technique [37]. Silver nitrate (AgNO_3_) (Sigma-Aldrich, Saint Louis, MO, USA) and ethylenediaminetetraacetic acid (EDTA) (Sigma-Aldrich, Saint Louis, MO, USA) were used in combination with the most effective dendron tested ArCO_2_G_2_(SNMe_3_I)_4_ (**2**). The concentrations used to treat biofilm formation (Section 2.3) ranged from 1 to 32 mg/L for ArCO_2_G_2_(SNMe_3_I)_4_ (**2**), from 0.5 to 16 mg/L for AgNO_3_ and from 8 to 256 mg/L for EDTA. For stablished biofilms (Section 2.4), the concentrations ranged from 32 to 1024 mg/L for ArCO_2_G_2_(SNMe_3_I)_4_ (**2**), from 32 to 1048 mg/L for AgNO_3_ and from 16 to 512 mg/L for EDTA. After treatments, resazurin assay and drop plate method were used as described in Section 2.6 and Section 2.7 to determine the MBIC, MBDC, MFC and MBEC values.

### 2.6. Resazurin Assay

Resazurin (Sigma-Aldrich) solution at 0.01% (*w*/*v*) was prepared in sterile distilled water [9,38,39]. The solution was filtered using a 0.22-μm-pore-size filter, and conserved at 4 °C. After treatment and incubation times, each well was washed with PBS, and then 20 μL resazurin was added to each well containing 100 μL of PBS. Plates were incubated in the dark at 37 °C for 24 h. Absorbance was measured at 570 and 600 nm in a microplate reader (Epoch^TM^, BioTek Instruments Inc, Winooski, VT, USA). 

These assays were used to determine the MBIC values (in the biofilm formation experiments) and the MBDC values (in the established biofilm experiments). 

### 2.7. Drop Plate Method

For these assays, biofilms were scraped and 5 μL suspensions were transferred onto Chloramphenicol-Sabouraud agar plates (Scharlab, Barcelona, Spain). All plates were incubated for 24 h at 37 °C. However, they were incubated for additional 24 h at 37 °C and let at room temperature for other 48 h when growth was not observed. 

The MFC values (in the biofilm formation experiments) and the MBEC values (in the established biofilm experiments) were obtained by the drop plate method [40]. These values were determined at concentrations where growth was not observed.

### 2.8. Cytotoxicity Evaluation

The cytotoxicity was studied for the most efficient dendron and combinations against this strain of *C. albicans.* The cytotoxicity was evaluated using HeLa cells (ATCC^®^ CCL-2TM). Assays were performed in 24-well plates (NUNCTM) in Dulbecco’s Modified Eagle Medium supplemented with 10% foetal bovine serum (Sigma-Aldrich Ltd.) and 1% antibiotic mix: 10.000 U penicillin, 10 mg streptomycin and 25 μg AmB per mL (Sigma-Aldrich Ltd.). Cells were seeded at a density of 1 × 10^4^ cells/well in 500 μL of fresh medium. Plates were incubated at 37 °C in a 5% CO_2_ atmosphere for 5 days, until a confluent monolayer was formed. At the end of incubation time, the medium was replaced by 400 uL of the serial concentrations of each compound (dendron, AgNO_3_ and EDTA) or combinations of compounds (dendron-AgNO_3_ and dendron-EDTA), diluted in fresh medium. Control wells received 400 μL of fresh medium. After 48 h of incubation, the culture medium was discarded, wells were washed three times with PBS and 500 μL of medium were added to each well. To evaluate the cytotoxicity, each well received 50 μL of microculture tetrazolium (MTT, 5 mg/mL) (Sigma-Aldrich Ltd.) and plates were incubated for 4 h at 37 °C. Subsequently, medium was discarded and 500 μL of dimethyl sulfoxide were added to dissolve formazan crystals. Absorbance values were recorded in a microplate absorbance reader at 570 nm (BioTek Instruments Inc. Model: ELX 800). Experiments were performed in triplicate and repeated at least twice. 

Viability percentage was calculated as OD_570_ compound/OD_570_ control × 100 (control wells were considered 100%).Reduction in viability percentages <10% were considered non-cytotoxic, values between 10–25% were considered low cytotoxicity, and values between 25–40% were considered moderate cytotoxicity levels [41]. 

### 2.9. Ultrastructural Study

The cell damage in *C. albicans* biofilm structures was evaluate using scanning electron microscopy (SEM). Sublethal concentrations were tested, and untreated controls were included. Morphological differences between biofilms treated with ArCO_2_G_2_(SNMe_3_I)_4_ (**2**), and the dendron in combination with EDTA and AgNO_3_ were evaluated. Both the biofilm formation and established biofilm treatment samples were studied. 

*C. albicans* was grown on a glass coverslip as explained above for the biofilm assays. Milloning’s solution containing 2% glutaraldehyde was used to fix the biofilm. In this case, 24 h later, cells were washed in Milloning’s solution with glucose (0.5%) [9]. Each glass coverslip was dehydrated in graded series of ethanol (30%–50%–70%–95%–100%), and then in anhydrous acetone, incubating for 7 min each. A Polaron CPD7501 critical-point drying system, and 200 Å gold-palladium sputter coating by Polaron E5400 were used on the samples. SEM was performed in a Zeiss DSM 950 microscope at 5–15 kV. The samples were prepared in triplicate, and different fields were observed for each sample. 

### 2.10. Statistical Analysis

All experiments were performed in triplicate. Two-way analysis of variance (ANOVA) and post-hoc comparisons with Dunnett’s multiple comparison tests were used to evaluate significance of differences. Statistical significance was defined as *p* < 0.05. Analysis was carried out using GraphPad Prism 9^®^ (GraphPad Software, San Diego, CA, USA).

## 3. Results and Discussion

### 3.1. Effective Prevention of C. albicans Biofilm Formation

Cationic carbosilane systems of different topology (spherical, wedge and bow-tie) have been describes as a antibacterial compound with a broad spectrum of action due to the presence of the ammonium groups on the surface (positive charge). The dendritic compounds used in this work are cationic carbosilane dendrons derived from 4-phenylbutyric acid. The principal differences between them are their generation that determine the number of ammonium groups on the surface. The increase in dendritic generation determines the hydrophilic/hydrophilic balance of the generated systems, which increases with increasing generation and determines how they interact with the cell membrane [17]. These dendrons present a 4-phenylbutyric acid (PBA) moiety at the focal point. PBA is drug used for urea cycle disorders and its effects on microorganisms are poorly studied [42]. However, some assays have shown that it can reduce *Shigella* infection in vivo using animal models [43], reduce colonization by *Salmonella enterica* serovar *Typhimurium* [44], inhibit the growth of *Helicobacter pylori* and *Escherichia coli* [45] and reduce herpes simplex virus infection [46]; among others. One of their advantages is also their solubility in water, thus overcome the issues generated by some antifungals that have the disadvantage of poor solubility [47].

The results obtained indicate that these compounds showed an antifungal activity against *C. albicans* and exerted activity preventing biofilm formation (Table 1) and the second generation dendrimer dendron ArCO_2_G_2_(SNMe_3_I)_4_ (**2**) with 4 positive charges was the most active molecule preventing *C. albicans* biofilm formation among all dendron tested. 

**Figure 2:**
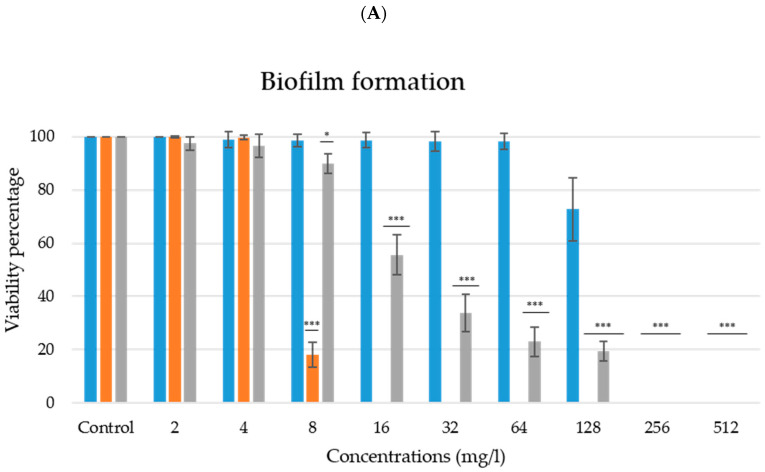

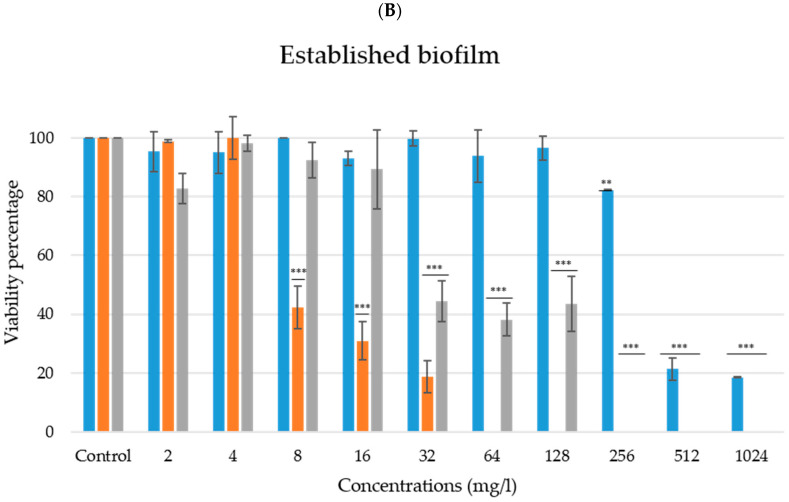
ArCO_2_G_1_(SNMe_3_I)_2_ (**1**)—blue, ArCO_2_G_2_(SNMe_3_I)_4_ (**2**)—orange, ArCO_2_G_3_(SNMe_3_I)_8_ (**3**)—grey. (**A**) Antifungal activity and inhibition of the formation of *C. albicans* biofilm in the biofilm formation experiments, (**B**) Antibiofilm activity against stablished biofilms in the established biofilm experiments. *p* values: * *p* < 0.05, ** *p* < 0.01, *** *p* < 0.001.

For *C. albicans* CECT 1002, the MBIC and MFC values were 16 mg/L using ArCO_2_G_2_(SNMe_3_I)_4_ (**2**); however, the ArCO_2_G_1_(SNMe_3_I)_2_ (**1**) and ArCO_2_G_3_(SNMe_3_I)_8_ (**3**) dendrons had an MBIC and MFC value of 256 mg/L (Table 1). Even though they showed the same MBIC value, a reduction to 55.55 ± 7.48% in viability percentage was observed with 16 mg/L concentration when the dendron ArCO_2_G_3_(SNMe_3_I)_8_ (**3**) was used, and 98.62 ± 2.75% when the dendron ArCO_2_G_1_(SNMe_3_I)_2_ (**1**) (*p* < 0.001) (Figure 2A). In addition, *Candida* viability remained at 98.06 ± 3.00% using ArCO_2_G_1_(SNMe_3_I)_2_ (**1**) at 64 mg/L (*p* > 0.05) while it was reduced to 23.06 ± 5.56% (*p* < 0.001) when ArCO_2_G_3_(SNMe_3_I)_8_ (**3**) was tested at 64 mg/L (Figure 2). However, we did not observe a gradual increasing antifungal activity with increasing generation, because the most active compound was ArCO_2_G_2_(SNMe_3_I)_4_ (**2**) (generation 2), that reduced viability completely. This is in agreement with previously results described in the literature due to the importance of the hydrophilicity-hydrophobicity balance in the dendritic skeleton that determine the efficiency-generation relationship [17,48]. In addition, higher generations also showed a greater cytotoxicity [17].

Our data demonstrated that ArCO_2_G_2_(SNMe_3_I)_4_ (**2**) antifungal activity (MBIC and MFC = 16 mg/L) was similar to the in vitro values required for some antifungals commonly used for *C. albicans*, such as amphotericin [49]. Some antifungals, such as fluconazole, facilitates the development of resistance due fungistatic conditions [50]. However, dendritic systems have been previously described to not generate resistance [17]. In our case, the ArCO_2_G_2_(SNMe_3_I)_4_ (**2**) antifungal and antibiofilm activity may be due to the mode of action proposed for these types of polycationic molecules related to their ability to interact with and penetrate the negatively charged membranes. This interaction removes divalent cations that maintain membrane integrity, modifying membrane permeability and leading to its disintegration [17,51,52]. Dendritic molecules target different cell structures and cause several metabolic disruptions, such as membrane disruption, membrane depolarization or cell cycle arrest [11,53]. In our previous studies, this effect was observed by SEM, including the formation of pores in the membrane of *C. albicans* [9]. These concurrent damages cause *Candida* cells to have decreased viability and reduce their ability to withstand the effects produced by treatments [54]. In contrast, the mode of action of antifungals is by targeting specific molecules or cell structures. 

### 3.2. Antibiofilm Activity against Established Biofilms 

*C. albicans* has the potential to form biofilms in different surfaces, and this fact increases the resistance of these fungi to antifungals. As anticipated by the results obtained in the study of the dendrons activity in the biofilm formation, the results obtained in the treatment of already formed biofilms showed that the activity of ArCO_2_G_3_(SNMe_3_I)_8_ (**3**) and ArCO_2_G_1_(SNMe_3_I)_2_ (**1**) dendrons was less effective at damaging the established biofilms (MBDC value of 256 mg/L and >1024 mg/L, respectively) when compared to the ArCO_2_G_2_(SNMe_3_I)_4_ (**2**) dendron which damaged the biofilm at a concentration of 64 mg/L (MBDC) (Table 1) (*p* < 0.001). In addition, viability was reduced to 50% at a concentration of 8 mg/L (*p* < 0.001) (Figure 2B). As expected, these values were all higher than our MBIC values; however, they were able to damage biofilms. In contrast, we did not reach the MBEC values for any of the dendrons tested, as growth was observed at all the concentrations in the drop plate method. We found cells that persisted when cell suspensions were plated on agar plates [55,56]. Similar results were described in our previous study. A spherical dendrimer of generation 0 that containing a tetrasiloxane ([SiO]_4_) core four -NMe_3_^+^ terminal groups on the periphery (named BDSQ024) showed a MFC of 32 mg/L and was not able to eliminate 100% of the viable cells from the stablished biofilm of *C. albicans* (MBEC was not determined) [9]. Despite not being a dendron tested in that study, the dendron ArCO_2_G_2_(SNMe_3_I)_4_ (**2**) shares some characteristics with the BDSQ024 dendrimer, including the presence of 4 NMe^3+^ groups on its surface (cationic molecule) and Si and S in its structure. Even though the rest of the molecular structure is completely different, knowing the importance of the presence of a certain number of exposed charges could be key when developing new compounds. It is also important to remark, that these dendritic molecules may not generate drug resistance in microbial cells. This may be due to their positive charge that interacts with the negatively charged cell surface and not with a specific organelle, protein or other molecule [53]. Further studies would be conducted by our research group to determine their exact mode of action. 

### 3.3. Combined Activity of ArCO_2_G_2_(SNMe_3_I)_4_ (**2**) and AgNO_3_

The synergistic effect between AgNO_3_ and ArCO_2_G_2_(SNMe_3_I)_4_ (**2**), the most effective dendron, was evaluated to determine if combination therapy might improve its efficacy and reduce the effective concentration. In the biofilm formation experiments, it was observed that the combination of both compounds exhibited a strong activity preventing biofilm formation. For instance, the combination 8:4 mg/L (ArCO_2_G_2_(SNMe_3_I)_4_ (**2**): AgNO_3_) reduced the *C. albicans* cell viability to 11.61 ± 2.58%. A reduction of 51.13 ± 12.7% was achieved when combining a lower concentration of AgNO_3_ (0.5 mg/L) with 8 mg/L of ArCO_2_G_2_(SNMe_3_I)_4_ (**2**) (Table 2). However, the MFC values obtained for the compounds in individual assays were not reduced in any case when combinations were analysed. 

Regarding the effect of the compounds in the already formed biofilm experiments (established biofilms), AgNO_3_ tested independently (Table 2) was able to damage the biofilm cells. However, it did not manage to disrupt and completely eliminate the viability of the biofilm cells at the concentration of 1024 mg/L (MBEC) (same as observed with ArCO_2_G_2_(SNMe_3_I)_4_ (**2**)). The MFC and MBEC values included in Table 2 represent the combinations of (ArCO_2_G_2_(SNMe_3_I)_4_ **2**)-AgNO_3_ and (**2**)-EDTA that eradicated cell viability.

Silver has extracellular and intracellular binding properties. Therefore, this molecule has the ability to bind to the microbial wall and, once it enters the cell, to bind to and damage proteins, genetic material or denature cytoplasmic enzymes, among other effects [21,22]. Additionally, this type of dendron also binds to the negatively charged cell membranes due to electrostatic interactions causing destabilization of the membrane, enhancing its effectiveness against established biofilms. Indeed, the combination therapy was able to completely kill cells and eradicate an established biofilm at a much lower concentration of 32 mg/L of AgNO_3_ and 32 mg/L of ArCO_2_G_2_(SNMe_3_I)_4_ (**2**) (Table 2). The anti-microbial effect of silver has been reported previously by other authors against planktonic cells and biofilms [21,27,57,58]. 

### 3.4. Combined Activity of ArCO_2_G_2_(SNMe_3_I)_4_ (**2**) and EDTA 

The synergistic effect between EDTA and ArCO_2_G_2_(SNMe_3_I)_4_ (**2**) was also evaluated. In individual biofilm formation treatment experiments, ArCO_2_G_2_(SNMe_3_I)_4_ (**2**) had a MFC of 16 mg/L and the EDTA failed to kill 100% of the cells at any concentration tested. The results obtained in the combination of ArCO_2_G_2_(SNMe_3_I)_4_ (**2**) and EDTA indicate that it was possible to reduce the MFC value for both compounds using 8 mg/L and 256 mg/L, respectively. In addition, using less concentrated EDTA (32 mg/L) it was possible to reduce *C. albicans* viability to 24.08 ± 7.32% (Table 2).

Regarding the established biofilm treatment, promising results were also found when EDTA was studied in combination with ArCO_2_G_2_(SNMe_3_I)_4_ (**2**). In this case, we again managed to eradicate the viability of the biofilm cells completely; however, higher concentration of ArCO_2_G_2_(SNMe_3_I)_4_ (**2**) compound was required for established biofilms (Table 2). Even though, we verified that using a combination of 32:16 (ArCO_2_G_2_(SNMe_3_I)_4_ (**2**)-EDTA) was able to reduce viability to 42.59 ± 3.40% of the cells forming the biofilm (Table 2). EDTA is a chelating agent that is used as an aseptic agent, enhancing the activity of other compounds, even in wound dressings for the control of microorganisms [31]. In yeast, it is believed that the mechanism of action presented by EDTA is related to the formation of complexes with certain ions (Mg^2+^ and Ca^2+^), inhibiting cell growth and causing its death [31]. In vitro eradication of *Candida* spp. biofilms using minocycline-EDTA-ethanol antimicrobial catheter lock solution has been reported [59,60].

### 3.5. Cytotoxicity

The cytotoxicity of the ArCO_2_G_2_(SNMe_3_I)_4_ (**2**) dendron and the combinations tested in this research were studied in Hela cells. Our data indicated that 16 mg/L ArCO_2_G_2_(SNMe_3_I)_4_ (**2**), the MBIC and MFC values for this dendron, showed high cytotoxicity at 48 h treatment. On the other hand, AgNO_3_ was highly cytotoxic at the concentrations ranging from 4 to 256 mg/L (MBIC 8 mg/L and MFC 8-16 mg/L), and EDTA showed no cytotoxicity at the concentrations ranging from 16 to 128 mg/L, but low cytotoxicity was observed at 256 mg/L EDTA (MBIC and MFC > 1024 mg/L). Therefore, it is shown that AgNO_3_ cytotoxicity was higher than EDTA cytotoxicity. 

The cytotoxic effects were also studied on compounds and concentrations used in combinations that affected the biofilm formation and established biofilm treatments (Table 3). In the biofilm formation-treatment, our data indicated that the effective combination ArCO_2_G_2_(SNMe_3_I)_4_ (**2**):EDTA (8:256) showed cytotoxicity (MFC combination value). In addition, the combination ArCO_2_G_2_(SNMe_3_I)_4_ (**2**):EDTA (8:32), that reduced biofilm viability to 24.08 ± 7.32%, and the combination ArCO_2_G_2_(SNMe_3_I)_4_ (**2**):AgNO_3_ (8:0.5), that reduced biofilm viability to 51.13 ± 12.7%, showed moderate and high cytotoxicity, respectively. We remark that combinations achieved a reduction in compound concentrations with an increase in activity and a significant reduction in cell cytotoxicity.

Related to established biofilm treatment values, the effective combinations that eliminated biofilm cells (MBEC combination value), ArCO_2_G_2_(SNMe_3_I)_4_ (**2**):EDTA (256:16) and ArCO_2_G_2_(SNMe_3_I)_4_ (**2**):AgNO_3_ (32:32), were cytotoxic against the cell line. Despite these cytotoxicity results, new molecules, such as silver nanoparticles or new combinations of EDTA with another similar dendron, could improve the cytotoxicity of the compounds, making them not only effective, but also less cytotoxic. In addition, these combinations could be completely useful for any other application that does not require direct human application, such as surface functionalization; and application in sanitizing or medical solutions (such as lotions).

### 3.6. Alterations Produced in C. albicans Cells on Biofilms Due to ArCO_2_G_2_(SNMe_3_I)_4_ (**2**) Activity

**Figure 3:**
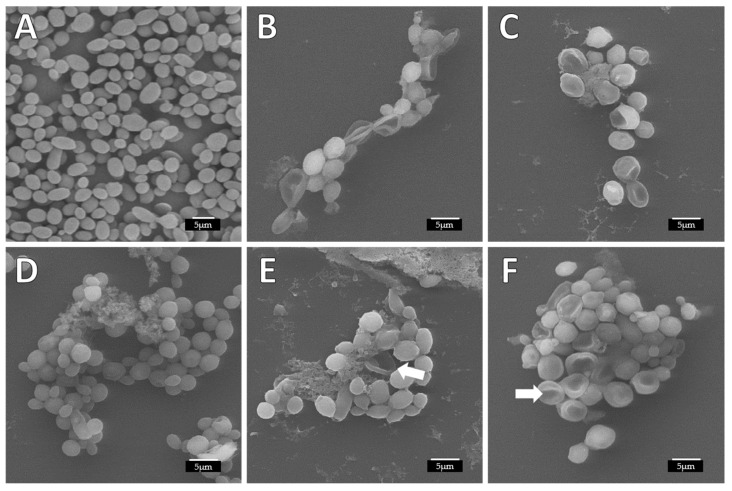
Alterations produced in *C. albicans* biofilm structure and cell morphology during the biofilm formation treatment observed by SEM. (**A**) Untreated; (**B**) ArCO_2_G_2_(SNMe_3_I)_4_ (**2**) dendron alone to 8 mg/L (**C**) AgNO_3_ alone to 8 mg/L; (**D**) EDTA alone to 32 mg/L; (**E**) combination ArCO_2_G_2_(SNMe_3_I)_4_ (**2**):AgNO_3_ to 8:4 mg/L; (**F**) combination ArCO_2_G_2_(SNMe_3_I)_4_ (**2**):EDTA to 8:32 mg/L (white arrow: collapsed cells).

The alterations due to the action of ArCO_2_G_2_(SNMe_3_I)_4_ (**2**) and combinations tested in the biofilm formation and stablished biofilm experiments against *C. albicans* biofilms were visualized and confirmed by SEM. In the biofilm formation treatment, we observed a perfectly homogeneous biofilm formation with layers of cells with smooth and regular walls in the untreated *C. albicans* controls (Figure 3A). However, in the treated samples with the different compounds the absence of a uniform layer and disrupted biofilm could be observed, as consequence of decreased and/or inhibition of biofilm formation Figure 3B–F). In Figure 3B, it was observed that the dendron ArCO_2_G_2_(SNMe_3_I)_4_ (**2**), damaged the biofilm cells and the biofilm structure was highly reduced, even at a concentration lower than its MBIC. In the case of AgNO_3_ (MBIC: 8 mg/L), the destruction of the cells was observed (Figure 3C), in contrast to EDTA, that showed no differences compared to the control (Figure 3D). Our data indicated that for combinations ArCO_2_G_2_(SNMe_3_I)_4_ (**2**):AgNO_3_ (8:4 mg/L) and ArCO_2_G_2_(SNMe_3_I)_4_ (**2**):EDTA (8–32 mg/L) the viability was approximately 25%. These results were verified by SEM, where cells appeared collapsed (Figure 3E,F. Arrows) at these sub-MBIC concentrations.

**Figure 4:**
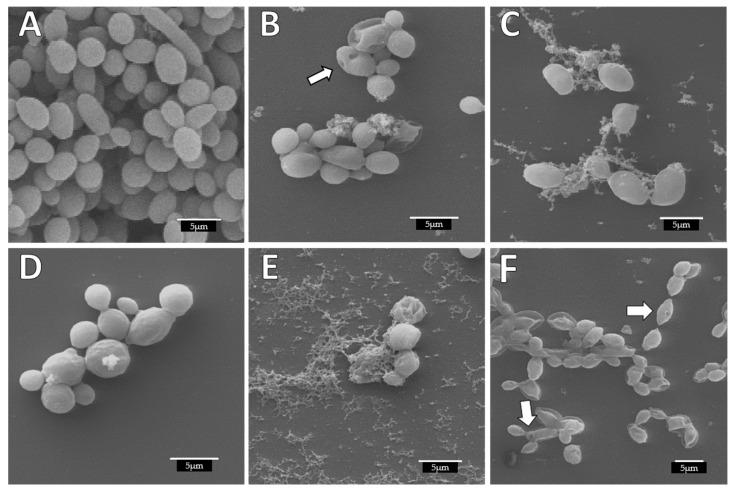
Alterations produced in *C. albicans* biofilm structure and cell morphology during the established biofilm-treatment observed by SEM. (**A**) Untreated; (**B**) 256 mg/L ArCO_2_G_2_(SNMe_3_I)_4_ (**2**) dendron; (**C**) 32 mg/L AgNO_3_; (**D**) 256 mg/L EDTA; (**E**) 32:32 mg/L combination ArCO_2_G_2_(SNMe_3_I)_4_ (**2**):AgNO_3_; (**F**) 256:32 mg/L combination ArCO_2_G_2_(SNMe_3_I)_4_ (**2**):EDTA (white arrow: collapsed cells).

The biofilms were also evaluated in the established biofilm. Again, differences were seen in the homogeneity of the control biofilm (Figure 4A) with respect to the treated *C. albicans* cells. The untreated biofilms displayed a uniform distribution, cells were attached to each other and showed a completely smooth membrane with a typical oval yeast shape. In contrast, treated biofilms previously formed with the compounds showed alterations (Figure 4B–F). The cellular damage caused by the dendron ArCO_2_G_2_(SNMe_3_I)_4_ (**2**) was seen as deformation on the cell wall and yeast morphology (Figure 4B, arrow: cells collapsed). These alterations were similar to the alterations previously described with the BDSQ024 dendrimer [9]. These biofilm disruptions and cell alterations has been observed in other studies [8]. In addition, we observed that yeast cells continue to attach to each other forming small groups of cells. This observation has been also reported [54]. In their study, they observed that biofilms treated with nanoparticles showed small multibranched groups of cells, usually formed by less than 10 cells. AgNO_3_ and EDTA showed less alterations (Figure 4C,D), although with EDTA treatment, roughness in the membrane was detected at high concentrations (256 mg/L) (Figure 3D). Our results indicated that ArCO_2_G_2_(SNMe_3_I)_4_ (**2**) dendron tested in combination with AgNO_3_ or EDTA, produced a synergistic effect that completely eradicated 100% of viable cells. This destruction was reflected in Figure 3E, where abnormal cell morphology and significant cellular deterioration was observed. In the case of ArCO_2_G_2_(SNMe_3_I)_4_ (**2**):EDTA combination, a more compact but clearly destroyed and altered biofilm was observed, including cells with certain perforations (Figure 4F. Arrows).

## 4. Conclusions

In the present study, we found an effective cationic carbosilane dendron ArCO_2_G_2_(SNMe_3_I)_4_ (**2**) with a 4-phenyl butyric (PBA) at the focal point and four ammonium groups on the surface groups, capable of preventing biofilm formation and damaging *Candida albicans* biofilms. The activity of this compound is comparable to some antifungal drugs and it could be used to control the nosocomial spread of this pathogen, especially in hospitalized patients. Therefore, we suggest that it may be used in healthcare, as a sanitizer solution for surfaces or a lotion to control skin infections. Additionally, we have managed to seriously damage an already formed biofilm, eradicating cell viability in its entirety using different combinations of dendron together with EDTA and AgNO_3_. These compounds could be also applied in the biomedical field with the objective of functionalized surfaces. Effective concentrations showed some cytotoxicity but the aim in future studies is to reduce cytotoxicity studying either new structures of the dendrons analysed in this study or other kind of dendrimers.

## Figures and Tables

**Table 1:** Antifungal activity against *C. albicans* CECT1002 of compounds **1**–**3** in biofilm formation (MBIC and MFC * (mg/L)) and in stablished biofilm (MBDC and MBEC * (mg/L)).

Dendrons	Biofilm Formation		Stablished Biofilm	
	MBIC (mg/L)	MFC * (mg/L)	MBDC (mg/L)	MBEC * (mg/L)
ArCO_2_G_1_(SNMe_3_I)_2_ (**1**)	256	256	>1024	BNE
ArCO_2_G_2_(SNMe_3_I)_4_ (**2**)	16	16	64	BNE
ArCO_2_G_3_(SNMe_3_I)_8_ (**3**)	256	256	256	BNE
AgNO_3_	8	8–16	32	BNE
EDTA	>512	>512	>1024	BNE

BNE: Biofilm not eradicated. * Drop plate method. MBIC: minimum biofilm inhibitory concentration. MFC: minimum fungicidal concentration. MBDC: minimum biofilm damaging concentrations. MBEC: minimum biofilm eradicating concentration.

**Table 2:** Viability percentage of cells forming *Candida* biofilms. The minimum fungicidal concentration (MFC) and minimum biofilm eradicating concentration (MBEC) values for the different combinations of ArCO_2_G_2_(SNMe_3_I)_4_ (**2**) -AgNO3 and ArCO_2_G_2_(SNMe_3_I)_4_ (**2**) -EDTA are indicated.

**Biofilm formation**	**ArCO_2_G_2_(SNMe_3_I)_4_** **(2) and AgNO_3_**			**ArCO_2_G_2_(SNMe_3_I)_4_** **(2) and EDTA**		
	(**2**):AgNO_3_ (mg/L)	Viability %	MFC *	(**2**):EDTA (mg/L)	Viability%	MFC *
	8:4	11.61 ± 2.58%	BNE	8:256 (MFC)	0.00 ± 0.00%	BE
	8:0.5	51.13 ± 12.7%	BNE	8:32	24.08 ± 7.32%	BNE
**Established biofilm**	**ArCO_2_G_2_(SNMe_3_I)_4_** **(2) and AgNO_3_**			**ArCO_2_G_2_(SNMe_3_I)_4_** **(2) and EDTA**		
	(**2**):AgNO_3_ (mg/L)	Viability %	MBEC *	(**2**):EDTA (mg/L)	Viability%	MBEC *
	32:32 (MBEC)	0 ± 0.00%	BE	256:16 (MBEC)	0 ± 0.00%	BE
				32:16	42.59 ± 3.4%	BNE

BNE: Biofilm not eradicated. BE: Biofilm eradicated. * Drop plate method.

**Table 3:** Viability percentage of HeLa cells treated with combinations.

Combination	Viability%
ArCO_2_G_2_(SNMe_3_I)_4_ (**2**):AgNO_3_ (32:32)	1.32 ± 0.63
ArCO_2_G_2_(SNMe_3_I)_4_ (**2**):AgNO_3_ (8:4)	1.72 ± 0.68
ArCO_2_G_2_(SNMe_3_I)_4_ (**2**):AgNO_3_ (8:0.5)	51.91 ± 3.73
ArCO_2_G_2_(SNMe_3_I)_4_ (**2**):EDTA (256:16)	1.73 ± 0.10
ArCO_2_G_2_(SNMe_3_I)_4_ (**2**):EDTA (32:16)	7.85 ± 0.21
ArCO_2_G_2_(SNMe_3_I)_4_ (**2**):EDTA (8:256)	56.46 ± 4.35
ArCO_2_G_2_(SNMe_3_I)_4_ (**2**):EDTA (8:32)	61.91 ± 3.24
